# Lateral Approach Versus Combined Lateral and Anteromedial Approach for Surgical Treatment of Terrible Triad of Elbow: A Meta-Analysis

**DOI:** 10.29252/beat-080102

**Published:** 2020-01

**Authors:** Mukesh Kumar Meena, Karmbeer Singh, Sanjay Meena, Chetan Kumbhare, Dushyant Chouhan

**Affiliations:** 1 *Department of Orthopedics, Lady Hardinge Medical College(LHMC) and Associated Hospitals, New Delhi, India* *.*

**Keywords:** Terrible triad of the elbow, Postoperative complications, Operative approaches

## Abstract

**Objective:**

To find out which surgical approach, optimize the functional outcomes and reduce the risk of complications in terrible triad of elbow".

**Methods:**

Medline, EMBASE, Cochrane Library, and Google Scholar were searched to identify relevant studies, which were included if they were retrospective or prospective in design, involved participants who had terrible triad of elbow (TTIE) that compared lateral approach (LA) with combined lateral and anteromedial approach (CML), and were published in English. Outcomes of interest were functional outcomes, complications, and operative time.

**Results:**

Four studies, involving 470 patients were included in the systematic review. Mean follow up after surgery was typically 24 to 30 months. We found significant more range of motion (ROM) of elbow in CML as compared to LA group (MD: -14.21, 95% CI: -21.13 to-7.29, *p*<0.00001). There was significant more forearm rotation in CML as compared to LA group (MD: -18.88, 95% CI: -32.35 to -5.40, *p*<0.00001). Mayo elbow performance score (MEPS) was significantly more in CML (MD: -3.31, 95% CI: -7.23 to 0.62, *p*=0.00001). Blood loss, operative time, VAS and complications were more in CML group; however, the difference was not significant. The heterogeneity of the study and synthesizing retrospective data were the primary limitations.

**Conclusion:**

Our analysis demonstrated that combined lateral and medial approach had significantly more elbow ROM and forearm rotation. The combined approach also had significantly more MEPS. However, using combined approach significantly increased the operative time.

## Introduction

The combination of elbow dislocation with both radial head and coronoid process fracture is notoriously challenging to treat and is termed “terrible triad” injury of the elbow (TTIE) [1]. This type of elbow injury typically occurs due to low or high energy falls onto an outstretched hand, which results in valgus and axial compression of the supinated forearm [2]. This leads to failure of the lateral collateral ligament complex (the medial collateral ligament may also fail), dislocation of the elbow, and consequent fracture of the radial head and coronoid process [2, 3].

As a result of these injuries, the elbow is left in an unstable state that invariably requires surgical intervention. Unfortunately, due to the complexity of injury, outcomes have traditionally been poor, with long-term complications including stiffness, pain, arthritis, and joint instability [4]. The aim of surgery in managing TTIE is the restoration of stability of the humeroulnar and humeroradial joints, thus facilitating early postoperative elbow motion to reduce likelihood of long-term joint stiffness or disability [3, 5]. Clearly, to optimize the chances of success, such surgery must adequately account for all three injury components of the terrible triad [3]. 

A number of studies comparing lateral approach (LA) with combined mediolateral approach (CML) have been conducted [6-9]. However, these studies were limited in sample size and quality of methodology, and failed to draw a definitive conclusion on which operative approach is optimal for terrible triad of elbow in reducing complications and improving prognosis. To provide a robust support for clinical decision, we conducted a meta-analysis to evaluate the efficacy of these two approaches in treatment of terrible triad of elbow.

*Search strategy*

We searched the following electronic databases for studies comparing LA to CML for the treatment of TTIE in adults including PubMed, Embase, OVID, the Cochrane Library, Web of Science and CNKI database. The key words used were “terrible triad of elbow”, “terrible triad”, “lateral approach for TTIE”, and “combined mediolateral approach for TTIE ". Articles were searched up to September 2018. Google Scholar was also searched to investigate potentially relevant literature. In addition, the reference lists of included studies and all related review articles were checked for additional trials, published or unpublished.

*Selection criteria*

Inclusion criteria were (i) Skeletally mature patients (older than 18 years old) diagnosed with TTIE, (ii) Lateral approach intervention compared to combined lateral and anteromedial approach for surgical treatment of terrible triad of elbow, (iii) Outcome of operative time, blood loss, Mayo elbow performance score (MEPS) visual analog score (VAS), range of motion (ROM) of elbow, forearm rotation and complications, and (iv) Study design of all types of study and the exclusion criteria of studies other than English language and animal studies.

*Data collection and analysis*

Data were extracted for all studies that met the inclusion criteria. For each study, 2 review authors (MK and KS) independently completed data extraction forms tailored to the requirements of this review. All disagreements were resolved by discussion between the 2 review authors. If consensus could not be made, a third review author (SM) was asked to complete the data extraction form and discuss the paper with the other 2 authors until consensus was reached.

*Statistical analysis*

 Heterogeneity test and effect value were determined. This study used Review Manager 5.3 software for meta-analysis. Risk ratios (RRs) were calculated for dichotomous variables in each study. Standardized mean difference (MD) was calculated for continuous variables, and an 95% confidence interval (CI) was determined for all effect sizes. Heterogeneity was analyzed using Chi-square tests before meta-analysis (*p*=0.05). If there was no heterogeneity (*p*≥0.05, I2<50%), a fixed-effect model was used. Otherwise (*p*<0.05), a random effect model was used. Sensitivity analysis was conducted by step-wise removal of data sets. Data sets causing significant changes in pooled results when removed were analyzed further to assess the reason. We then judged the results for stability and strength. If the heterogeneity was too large to analyze, descriptive analyses are presented

**Publication bias and Quality of studies**

Publication bias was analyzed using Begg and Egger tests. *P* value less than 0.05 was considered significant. The coauthors MK and KC, performed a study level independent risk of bias assessment using the modified Downs and Black Quality Assessment checklist.

*Main Search results*

**Fig. 1:**
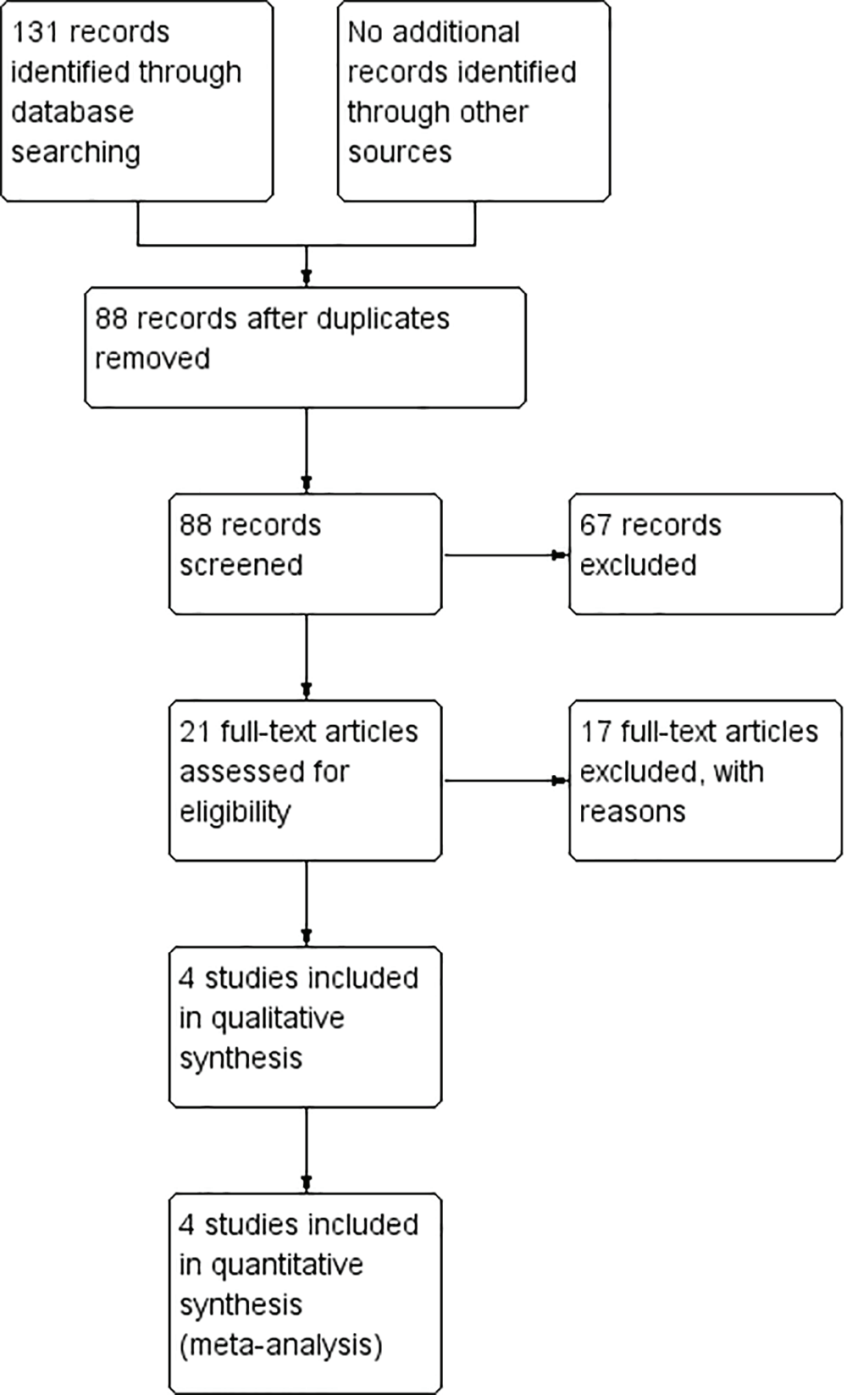
Study Searching Process

The literature search yielded 131 studies. Of these, 43 were duplicates and 67 did not match our inclusion criteria according to title and abstract assessment. No data was obtained from gray literature investigations or ongoing trials. For the remaining 21 studies, 17 did not meet the inclusion criteria after full-article assessment. Therefore, 4 studies with a total of 470 patients were included in this review. The search process is shown in Figure 1.

*Demographic characteristics*

Baseline characteristics LA and CML groups were similar. The main characteristics of the studies included in the systematic review were summarized in Table 1. The number of patients in the studies ranged from 26 to 217 (total= 470). All studies reporting information on patient sex included a majority (>50%) of male patients. Most studies reported that the mean age of patients was 33 to 50 years. The mean length of follow up ranged from 24-30 months only one study showed follow up data. 

**Table 1:** Characterstics of included studies

**1st Author** ** (year)**	**Patients number**	**Sex % male**	**Mean Age (range) years **	**Mean Follow-up (range), months**	**Radial Head Fracture Classification **	**Coronoid Fracture Classification b**
	Lateral⁄ CLAM	Lateral⁄ CLAM	Lateral⁄ CLAM	Lateral⁄ CLAM	Lateral⁄ CLAM	Lateral⁄ CLAM
Hong-Wei Chen (2016)^6^	12/ 14	66.67/ 64.28	37.12±3.1/ 36.62±2.5	NA	I:II:III :: 5:4:3 / I:II:III :: 3:8:3	I:II:III:IV :: 2:6:4:0 / I:II:III:IV :: 2:8:4:0
Hong-Wei Chen (2017)^7^	112 / 105	64.28 / 57.14	NA	NA	24:61:27 / 25:60:20	20:48:24:0 / 30:60:15:0
Chengwei Zhou (2018)^8^	28 / 32	64.29 / 71.86	41.8 ± 3.5 / 45.2 ± 5.2	26.1 (24-30)	3:25:0 / 2:30:0	1:3:24:0 / 2:8:22:0
Tao i(2018)^9^	88 /81	65.90 /65.43	39.57±3.84 / 40.81±3.55	NA	I:II:III::19:69:18, II or III- 69/ Type I-18, II or III- 63	Type I-32, II or III-56 / Type I-23, II or III-58

*Quality assessment*

The quality assessment using the modified Downs and Black Quality Assessment checklist was shown in Table 2.

**Table 2:** Modified Downs and Black scale for assessment of study quality

**1** ^st^ ** Author (year)**	**Reporting (11)**	**External Validity (3)**	**Internal Validity bias (7)**	**Internal Validity Confounding (6)**	**Power (1)**	**Total quality score (28)**
Hong-Wei Chen (2016)^6^	7	1	4	3	1	16
Hong-Wei Chen (2017)^7^	7	1	4	3	1	16
Chengwei Zhou (2018)^8^	8	1	3	3	1	16
Tao i (2018)^9^	7	1	3	3	1	15

*Range of flexion and extension at elbow*

**Fig. 2:**
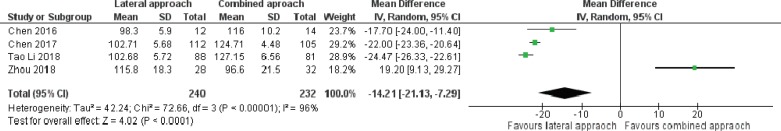
Range of Flexion and Extention at elbow

Four studies reported data on postoperative final follow-up of range flexion and extension of elbow including patients in the LA and CML groups. There was a significant difference in Rom between LA and CML (MD: -14.21, 95% CI: -21.13 to-7.29, *p*<0.00001) with statistical heterogeneity among studies (x^2^=72.66, *p*<0.0001, I^2^=96%, Figure 2).

*Rotation of forearm*

**Fig. 3:**
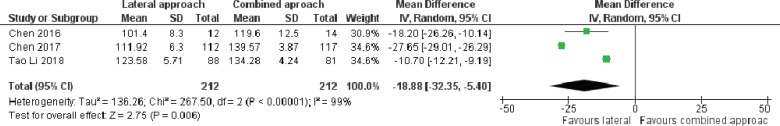
Rotation at forearm at final follow up

Three studies reported data on postoperative final follow up of range of rotation of forearm at final follow up including patients in the LA and CML groups. There was a significant difference regarding ROM between LA and CML groups (MD: -18.88, 95% CI: -32.35 to -5.40, *p*<0.00001) with statistical heterogeneity among studies (x^2^=267.5, *p*=0.006, I^2^=99%, Figure 3)

.

*MEPS*

**Fig. 4:**
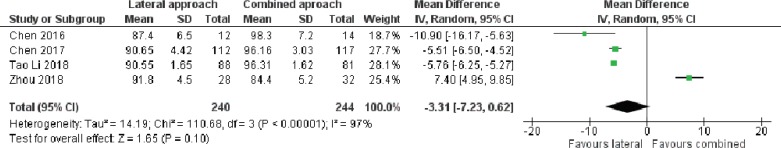
MEPS (Myo Elbow Performance Score) follow up

Four studies reported data on postoperative final follow up of MEPS including patients in the LA and CML groups. There was a significant difference for ROM between LA and CML groups (MD: -3.31, 95% CI: -7.23 to 0.62, *p*=0.00001) with statistically significant heterogeneity among studies (x^2^=110.68, *p*=0.10, I^2^=97%, Figure 4).

*Duration of operation time*

**Fig. 5:**
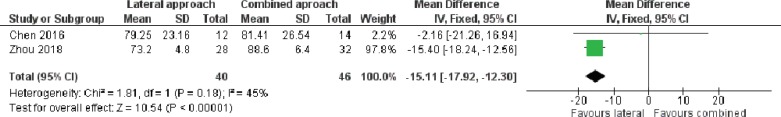
Duration of Operation Time

Two studies involving 195 fractures provided data on operation time. There was no significant heterogeneity among studies (x^2^=1.81, *p*=0.18, I^2^=45%), and the pooled outcome (operative time) and differred significantly between groups (MD: -15.11, 95%CI: -17.92 to -12.30 to, *p*<0.00001; Figure 5). 

*Blood loss*

**Fig. 6:**
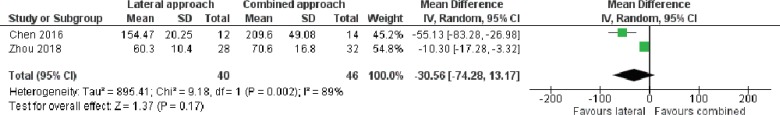
Study Blood Loss Data

Two studies involving 195 fractures provided data on blood loss. There was no significant heterogeneity among studies (x^2^=9.18, *p*=0.002, I^2^=89%), and the pooled outcome (blood loss) did differ significantly between groups (MD: -30.56, 95% CI: -74.28 to 13.17, *p*=0.17; Figure 6).

*VAS score*

**Fig. 7:**
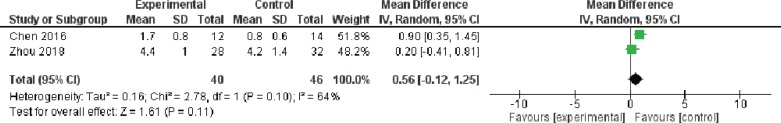
VAS score of the Study

Two studies involving 195 fractures provided data on VAS. There was a significant heterogeneity among studies (x^2^=2. 78, *p*=0.10, I^2^=64%), and the pooled outcome for VAS did not differ significantly between groups (MD: -0. 56, 95%CI: -0.12 to 1.25, *p*=0.11, Figure 7)

*Complications*

**Fig. 8:**
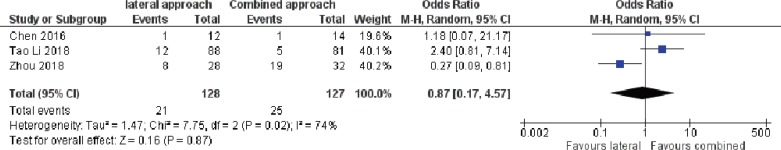
Data reported on Patient's Complication

Three studies reported data on complications including patients in the LA and CML groups. There was a significant heterogeneity among studies (x^2^=7.75, *p*=0.02, I^2^= 74) and the pooled outcome did not differ significantly between the two groups (OD: 0.87. 95% CI 0.17 to 4.57, *p*=0.87; Figure 8)

## Discussion

Terrible triad of elbow leads to extensive damage to ligamentous and osseous structures, which leads to acute elbow instability. Closed management is not an optimum treatment and they invariably require open reduction and internal fixation. Various approaches have been described for the same. The posteromedial approach can give access to both medial and lateral aspect of elbow joint, but has the disadvantages of long incision, extensive dissection, subcutaneous hematoma and risk of flap necrosis, so it has been obsoleted gradually [10-13]. 

Pugh *et al*., [14] showed good results with lateral approach in TTE. However, sometimes the soft tissue is too swollen, that a single lateral approach may be insufficient to obtain good surgical exposure and combined medial and lateral approach has to be used. However, there is still no consensus on the better approach. Chen *et al*., [6] demonstrated that combined approach leads to extended surgical exposure, fracture stability and reduced complication rate compared to lateral approach. To facilitate a clinical decision, we conducted a meta-analysis comparing lateral approach and combined approach for terrible triad of elbow

We found that the elbow range of motion, pronation-supination and MEPS were significantly more in the combined approach. MEPS is highly applied to evaluate disability of elbow fracture dislocation [15]. This suggests that lateral approach leads to poor stability as compared to combined approach, which leads to a poor functional outcome. MCL is one of the most important structures which provide valgus stability of elbow [16]. It is of paramount importance that MCL should be repaired in elbow fracture dislocation. Jeong *et al*., [17] demonstrated that restoration of damaged structures, including medial soft tissue structures gives excellent results based on MEPS in patients with terrible triad elbow. 

In terrible triad elbow, the lateral collateral ligament, MCL and anterior capsule are usually torn and soft tissues were injuries, all of which are important determinants in elbow stability [18]. Lateral approach alone may not give adequate exposure to repair all these important structures. Combined approach has advantages of providing both bone and soft tissue stability simultaneously allowing early exercise and improving early functional recovery [19]. This leads to significantly better MEPS in the combined group.

Combined approach may not be the best approach in terms of operative time, blood loss and wound healing as combined approach uses two approach which leads to increase in operative time. Our analysis also showed significantly more operative time in combined group. Our analysis also showed that combined lateral and medial approach led to more blood loss compared with lateral approach. This is expected since combined approach led to more extensive dissection as compared to lateral approach and is much more complicated surgical procedure. 

Chen demonstrated an increased healing time with combined approach when compared to lateral approach [6]. The increased healing time in turn may lead to delayed recovery. However, the combined approach is able to restore anterior and lateral lesions, as well as medial lesions and use of the lateral approach may not be able to remove all the small bone fragments [17]. The VAS score for combined approach is significantly lower in combined lateral and medical approaches.

During the TTE operation, lateral approaches are useful in addressing pathology at the radial head, coronoid process, and anterior and posterior capsules, while medial approach is effective in addressing ulnar nerve, the anterior and posterior capsules, and the coronoid process [20]. Although the combined lateral and medial approaches may be associated with longer recovery time, this approach was able to restore anterior and lateral lesions, as well as medial lesion in TTE tissue, and use of the lateral approach may not be able to remove all the small bone fragments [17].

Complication rate was higher in combined approach as compared with lateral approach. However, the difference did not reach a significant level. Our analysis demonstrated that combined lateral and medial approaches had significantly more elbow ROM and forearm rotation. The combined approach also had significantly more MEPS. However, using combined approach could significantly increase the operative time.

## Conflict of Interest:

None declared.
